# A Fast Method to Monitor Tyrosine Kinase Inhibitor Mechanisms

**DOI:** 10.1021/acs.jmedchem.4c02042

**Published:** 2024-11-08

**Authors:** Alejandro Fernández, Margarida Gairí, María Teresa González, Miquel Pons

**Affiliations:** † Biomolecular NMR Laboratory, Departament de Química Inorgànica i Orgànica, Universitat de Barcelona (UB), Baldiri Reixac 10-12, 08028 Barcelona. Spain; ‡ Centres Científics i Tecnològics de La Universitat de Barcelona (CCiTUB), Baldiri Reixac 10-12, 08028 Barcelona. Spain; § PhD Program in Biotechnology, Faculty of Pharmacy, Universitat de Barcelona (UB), 08028 Barcelona, Spain

## Abstract

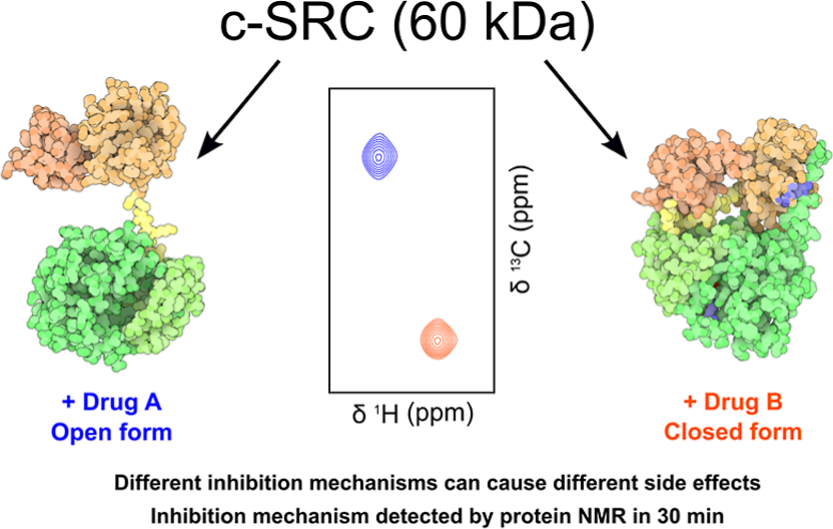

Methionine residues within the kinase domain of Src serve as unique NMR probes capable of distinguishing between distinct conformational states of full-length Src, including alternative drug-inhibited forms. This approach offers a rapid method to differentiate between various inhibition mechanisms at any stage of drug development, eliminating the need to resolve the structure of Src-drug complexes. Using selectively ^13^C-methyl-enriched methionine, spectra can be acquired in under an hour, while natural abundance spectra with comparable information are achievable within a few hours.

## Introduction

Tyrosine kinases represent a major portion of known oncogenes and cancer drug targets.^1,2^ More than 70 kinase inhibitors have been approved for therapeutic uses, mainly against cancer. Src is a prototypical nonreceptor tyrosine kinase and the leading member of the Src family of kinases (SFK). Despite being recognized as an important cancer target and the success of some inhibitors that also target other kinases in hematological cancers, Src inhibitors have failed so far in clinical trials for solid tumors. Thus, the development of Src-directed drugs remains an active field of research.

The domain structure of Src includes the enzymatically active kinase domain (KD) and the SH2, SH3, and Unique regulatory domains as well as a membrane-anchoring SH4 domain. The Src regulation mechanism involves the equilibrium between an open active state and a closed inactive form stabilized by the interaction of regulatory domains with the KD. The isolated KD is highly dynamic and can sample active and inactive conformations that are potential drug targets.^3^ The KD conformational ensemble and the kinase activity of Src are allosterically modulated by the interaction of the KD with the regulatory domains.^4,5^

The KD of Src and other protein kinases has two lobes, with the active site located in a cleft between them. The small N-terminal lobe (residues 270–341) is formed by an antiparallel β-sheet and an important regulatory αC-helix. A salt bridge between K298 in the β3 strand and E313 in the αC-helix is a prerequisite for the active state referred to as “αC-in” conformation. The small lobe contributes contact sites with the phosphate and adenine parts of ATP and interacts with ATP-competitive inhibitors. The inactive, autoinhibited conformation does not form this salt bridge and is called “αC-out”.

The large C-terminal lobe (residues 348–523) is mainly α-helical with short β-strands and contains a mobile activation segment that adopts an extended conformation in the active enzyme and a closed conformation in the inactive forms. The first residues of the activation segment contain a conserved DFG sequence in which the aspartic acid points to the active site in the active conformation (“DFG-Asp in”) and away from it in the inactive forms (“DFG-Asp out”). The SH2 regulatory domain interacts with phosphorylated Y530 in the tail that extends after the C-terminal lobe, and the SH3 domain makes extensive contacts with the N-lobe mediated by a polyproline region in the linker connecting the SH2 and KD.

Previous work has extensively used NMR to study the conformational equilibria of kinases, including the related Abelson tyrosine kinase and its allosteric opening and closing by ATP-site and myristoyl pocket inhibitors.^6−8^

Src inhibitors targeting the ATP binding site are classified into type 1, which lock the kinase in its active conformation, and type 2, which targets the inactive conformation. Inhibitor-induced conformational changes in the KD also influence the equilibrium between open and closed conformations, affecting the ability of the regulatory SH3 and SH2 domains to engage in protein–protein interaction hubs and mediate phosphotransfer-independent scaffolding activities.^9^ This additional inhibitory effect has been observed in recently reported Src inhibitor eCF506 but is absent in dasatinib, which locks Src in its active state.^10^

Currently, the type of inhibition and its effect on the regulatory domains must be experimentally determined by determining the X-ray structure of the complex with the full-length protein. NMR may provide much faster access to the inhibitory mechanism of candidate drugs in the discovery and optimization stages. Methyl-TROSY exploits the unique relaxation properties of CH_3_ groups to largely overcome the protein size barrier.^11^ The flexibility of the methionine side chains provides a potential additional narrowing mechanism facilitating the observation of Met-CH_3_ signals even in large nondeuterated proteins.^12^ Met-CH_3_ chemical shifts are highly sensitive to the spatial environment created by other residues in a 6 Å sphere,^13^ making them especially useful probes for studying large-scale conformational transitions associated with allosteric effects.^14,15^

N-terminally processed Src has only 10 methionine residues, all located in the KD. This small number of signals provides simple, interpretable spectra even for full-length Src (60 kDa). In contrast to alternative labeling strategies, such as the attachment of ^19^F tags that would also provide a small number of signals,^16^ methionine labeling is easier and leaves the protein intact.

Here, we show that Met-CH_3_ NMR signals are easily observable in the isolated KD as well as in full-length Src and they can monitor the response of the KD to natural regulatory mechanisms such as phosphorylation or the interaction with regulatory domains. Importantly, Met-CH_3_ also allows differentiation of inhibitors acting through distinct mechanisms.

## Results and Discussion

### Met-CH_3_ NMR Spectra Report on the Activation State of Src

The 10 methionine residues in the KD are strategically located in known functionally relevant regions. M317 is in the regulatory helix C and is part of the regulatory spine.^2^ M305 is located just before helix C and faces the ATP binding site. Rotation of helix C between the αC-in and αC-out is associated with the transition from an active to an inactive conformation. M369, M377, and M383 are in the E helix, which participates in SH2 binding. The CH_3_ of M377 is located about 5 Å from histidine 387, which is part of the catalytic loop and regulatory spine, and M377 and M383 are adjacent to residues A378 and E381, which are part of the αF pocket.^17^ M344 is in the hinge connecting the N- and C-lobes and is implicated in activation of catalysis.^18^ Thus, we decided to explore the capacity of Met-CH_3_ NMR to report on the distinct functional states of Src.

**Figure 1:**
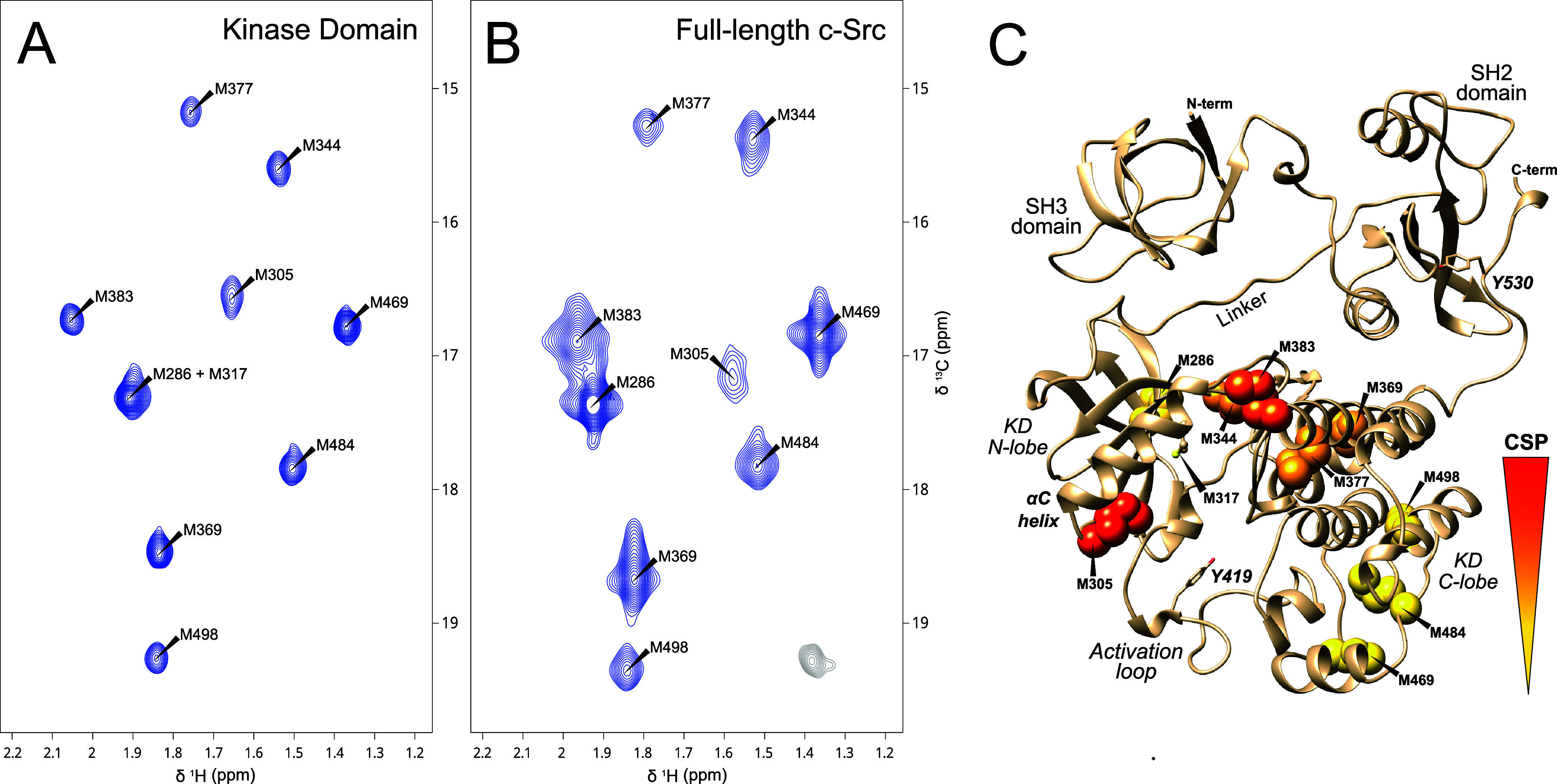
^1^H–^13^C HMQC correlation spectra of Met-^13^CH_3_-enriched Src KD (A) and full-length Src (B). A natural abundance signal from a nonmethionine methyl group is shown in gray contours. (C) The position of methionine groups is represented on the AlphaFold2 model of full-length Src with colors indicating the combined chemical shift perturbations (CSP) of Met-CH_3_ groups between the full-length protein and the isolated domain. CSP have been calculated as CSP = (0.5 (δ_H_)^2^ + (0.185δ_C_)^2^)^0.5^ based on the average variances of proton and carbon in the BioMagResBank.^19^ The protein concentration was ca. 100 μM in 20 mM sodium phosphate, pH = 7.5, 100 mM NaCl, and 0.01% NaN_3_ measured at 298 K in 3 mm tubes. KD and full-length spectra were measured at 600 and 800 MHz, respectively. Additional details are included in materials and experimental methods.

We expressed the isolated KD and full-length Src in a methionine auxotroph *Escherichia coli* strain grown in minimum media supplemented with ^13^CH_3_-labeled methionine. Methionine labeling is scramble-free.^20^ Well-resolved spectra with the expected number of signals were obtained for the KD (Figure 1A) and were assigned by mutating the individual methionine residues to leucine for residues located in α-helix regions or isoleucine for methionine residues in β-strands (Supplementary Figure S1). The signals from M286 and M317 overlap, but the M317 signal could be confirmed as a broad peak in the M286I KD mutant. The spectra of full-length Src (Figure 1B) have an extra signal from a natural abundance nonmethionine methyl.

Large signal shifts between the isolated KD and full-length Src were observed for M305, M383, and M344. The last two residues are in regions that contact with the regulatory SH3 domain though the SH2-KD linker and M305 is facing the activating loop connecting the two lobes of the KD (Figure 1C). These signal shifts provide a first indication that Met-CH_3_ are sensing the changes in the KD conformational states induced by the interaction with the regulatory domains.

### Met-CH_3_ NMR Spectra Reflect the Effect of Regulatory Tyrosine Phosphorylation

Because the equilibrium between the open and closed conformations of Src is naturally regulated by the interaction between the SH2 domain and phosphorylated Y530 in the C-terminal tail as well as by the phosphorylation of Y419 in the activation loop, we compared the spectra of phosphorylated and unphosphorylated forms of full-length Src, using the isolated KD, which lacks the regulatory domains, as a reference. Overnight incubation with ATP and magnesium chloride resulted in complete phosphorylation of Y419 and Y530.^21^

**Figure 2:**
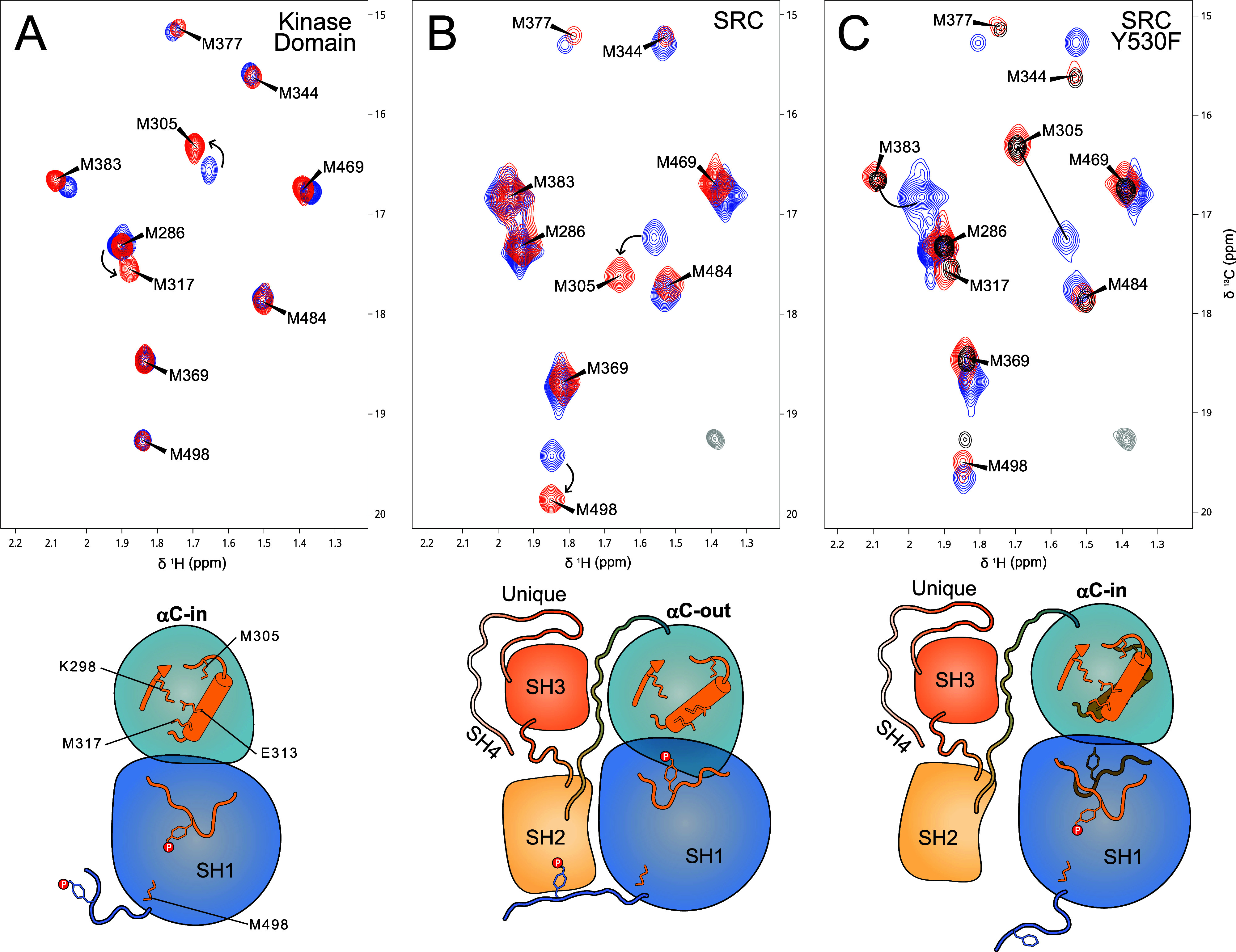
Met-CH_3_^1^H–^13^C HMQC correlation spectra of unphosphorylated (blue) and phosphorylated (red) Src KD (A), full-length Src (B), and Y530F mutant of full-length Src (C). M317 is on the same face of the C-helix as E313 that forms an essential salt bridge with K298 in the αC-in conformation and is absent in the αC-out state. M305 is sensing the activation state of Src. The αC-out conformation is observed when Y530 is phosphorylated, independent of the phosphorylation state of Y419. When the phosphorylation of Y530 is prevented in the Y530F mutant, phosphorylation of Y419 causes a transition from αC-out to αC-in. NMR conditions are the same as in Figure 1.

Figure 2 shows the effect of phosphorylation on the Met-CH_3_ spectra of the isolated KD, full-length wild type Src, and Y530F full-length Src where only Y419 can be phosphorylated.

Phosphorylation of the isolated KD (Figure 2A) causes significant chemical shift changes in M305, M317, and M383 and smaller changes in M469 and M377. M498, close to the C-terminus is completely unaffected. In contrast, phosphorylation of full-length Src (Figure 2B) causes large spectral changes involving the previous residues in addition to M498, M369, and M484 which are located close to the C-terminal inhibitory tail.

The effect of the phosphorylation of Y419 and Y530 can be distinguished using the Y530F mutant (Figure 2C). When unphosphorylated, the Met-CH_3_ spectrum of the Y530F mutant closely resembles that of wild-type Src. Phosphorylation of Y419 in the Y530F mutant leads to the complete release of interactions with the SH3 and SH2 regulatory domains, resulting in a spectrum resembling that of the isolated KD. In contrast, simultaneous phosphorylation of Y419 and Y530 retains the closed conformation. Thus, phosphorylation of Y419 and Y530 has opposite effects on Src activation, but the interaction of pY530 with the SH2 domain predominates.

**Figure 3:**
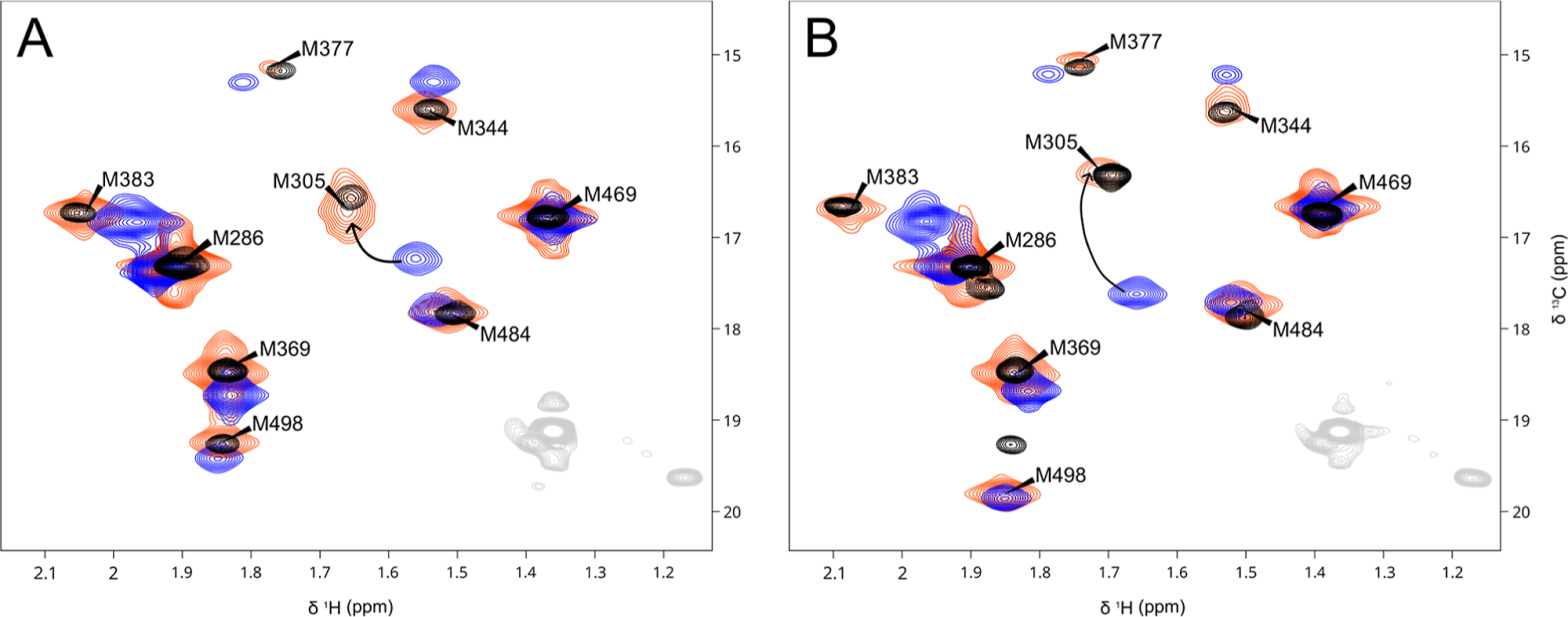
Met-CH_3_^1^H–^13^C HMQC correlation spectra of unphosphorylated (A) and phosphorylated (B) free Src (blue), VSL12 complex (red), and isolated KD (black). Natural abundance signals from Src and VSL12 peptide are shown in light gray. NMR conditions are the same as in Figure 1.

**Figure 4:**
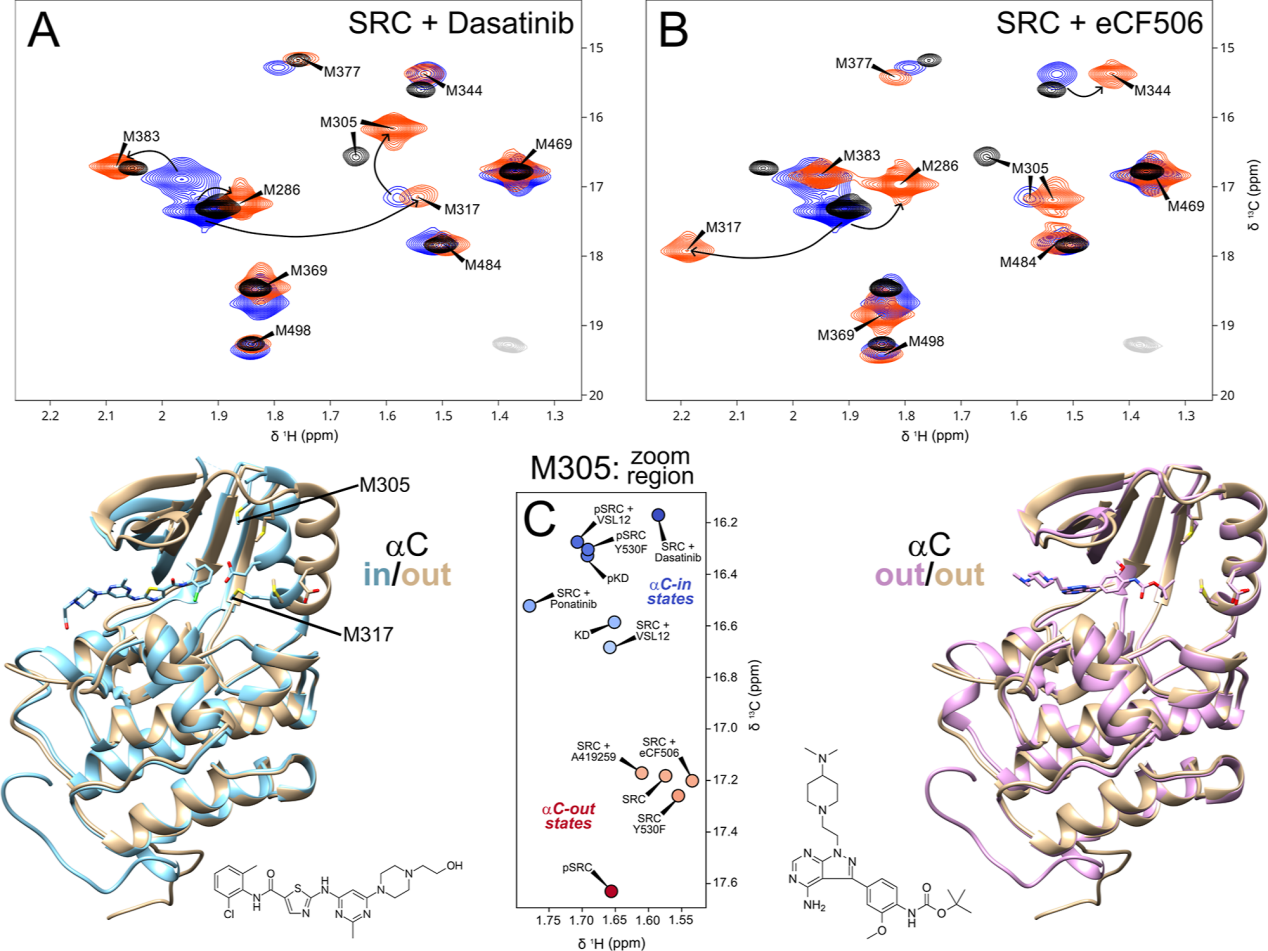
Met-CH_3_^1^H–^13^C HMQC correlation spectra of unphosphorylated full-length Src in the absence (blue) and in the presence (red) of dasatininb (A) and eCF506 (B) compared to that of the free KD (black). Natural abundance signals from Src are shown in light gray. The models below show the KDs of the autoinhibited form of Src (PBD 2SRC, brown), the eCF506 complex (PDB 7NG7, pink), and the dasatinib complex (PDB 3G5D, blue). (C) Schematic representation of the M305 peak in the different systems studied in this work. The label’s colors are assigned automatically in a blue to red scale according to the ^13^C chemical shift. NMR conditions are the same as in Figure 1.

The ^13^C chemical shift of M305 remains well-resolved and moves approximately 1 ppm, depending on the activation state of the kinase. Thus, M305 could be a reporter of the activation state of Src, specifically in relationship with the conformation of the αC-helix. This idea was confirmed when the active or inactive forms were induced by the interaction with ligands (Figures 3 and 4).

### Met-CH_3_ NMR Spectra Reflect the Effect of a Competitive Ligand Binding to the SH3 Regulatory Domain

In addition to changes in the phosphorylation state, Src activation can be achieved by competitive interactions with the regulatory domains. Therefore, we examined the effect of polyproline peptide VSL12, which binds with high affinity to the SH3 domain and prevents its interaction with the KD through the KD-SH2 linker.

Figure 3 compares the spectra of phosphorylated and unphosphorylated full-length Src in the presence or absence of an excess of polyproline peptide. The chemical shifts of the CH_3_ groups of M305, M344, M377, and M383 in the presence of the polyproline peptide are nearly identical to those observed in the isolated KD, indicating a complete local release of the SH3-KD interaction, in both the phosphorylated and unphosphorylated proteins. The chemical shift of the CH_3_ signal of M489 in the unphosphorylated protein is also identical with that of the isolated KD in the presence of the peptide. However, in the phosphorylated protein the signal of this residue does not change in the presence of the peptide, indicating that the SH2-pY530 interaction (sensed by the M498 chemical shift) is maintained. This is consistent with the canonical model in which the SH3 domain acts as a clamp with broadly distributed interactions while the SH2 domain acts as a latch stabilizing the closed conformation.^22^

### Met-CH_3_ NMR Spectra Differentiate between Distinct Inhibitory Mechanisms by ATP Competitive Drugs

Having demonstrated the sensitivity of Met-CH_3_ NMR spectra to the conformational plasticity of the KD in response to various natural regulatory processes, such as phosphorylation or competitive binding to regulatory domains, we next explored the response to two competitive Src inhibitors known to act through different mechanisms.

Dasatinib is a dual Src/Abl inhibitor that is currently used to treat BCR-Abl positive leukemias.^23^ The recently developed eCF506 inhibitor exhibits similar nanomolar affinity for Src but requires concentrations 3 orders of magnitude higher to inhibit Abl.^10^ Interestingly, dasatinib and eCF506 inhibit Src through entirely different mechanisms. The X-ray structure of the eCF506-Src KD complex reveals that the KD is locked in its inactive state, in contrast to dasatinib, which blocks Src in its active state (Figure 4).^10^ The inactive and active states adopt the αC-out and αC-in conformations, respectively, meaning that the two drugs are expected to induce distinct changes in the methionine methyl NMR spectra.

Figure 4A,B presents the Met-CH_3_ spectra of full-length Src in the presence of dasatinib and eCF506, respectively. The two drugs cause significant and distinct perturbations in the Met-CH_3_ signals. Full-length M317L and M305L mutants were used to confirm the assignments of signals with large perturbations (Supplementary Figure S2). M305 and M317 in helix C are the most affected signals. M317, being in direct contact with the drugs, exhibits large drug-specific chemical shifts. In contrast, M305, a reliable reporter of the activation state, clearly differentiates the inhibitory modes of the two drugs. Figure 4C compares the position of the M305 cross-peak in all of the systems studied. ^13^C chemical shifts are correlated with the activation state.

The lowest-field position (17.6 ppm) corresponds to the autoinhibited pSrc. The chemical shift of the M305 methyl group in the eCF506 complex is like that of unphosphorylated Src, which was previously shown by small-angle X-ray scattering (SAXS) to be 85% in the closed conformation.^24^ The highest-field position (16.1 ppm) corresponds to the dasatinib complex, where Src is locked in its active state. Other forms of Src with structural features of the active state also exhibit high-field ^13^C chemical shifts for M305, including the VSL12 complex (which disrupts the SH3-KD interaction), the phosphorylated Y530F mutant (where the SH2-KD interaction is not possible), and the isolated KD, where the SH3 and SH2 domains are absent. Thus, M305 ^13^C chemical shifts distinguish between the active/open conformation and the inactive/closed conformations of Src.

To test the general applicability of M305 as a probe for the inhibitory mechanisms of ATP competitive drugs, we tested two additional compounds: A419259^27,28^ and ponatinib.^25,26^

While there is no X-ray structure of A419259 in complex with Src, the structures of the complexes with other SFKs (Fgr, PDB: 7UY0 and Hck, PDB: 3VS3) show that the two proteins adopt a closed conformation lacking the salt bridge between E313 and K278 (αC-out), similar to that of autoinhibited Src or the Src-eCF506 complex. Consistently, the M305 ^13^C chemical shift clearly indicates that A419259 induces a closed conformation in Src, akin to the conformation induced by eCF506 (Figures 4C, S3).

The X-ray structure of the ponatinib-Src complex (PDB 7WF5) reveals a salt bridge between E313 and K278 (αC-in), like that in the dasatinib complex. However, it differs from dasatinib in that the aspartic acid side chain in the DFG conserved sequence points away from the active site (DFG-out). The ^13^C chemical shift of M305 of the ponatinib and dasatinib complexes are both in the αC-in region (Figures 4C, S3), confirming that M305 effectively reports on the activation state sensed by the C-helix.

### Src Regulation: KD Conformational Plasticity and Interaction with SH3/SH2 Domains

Src regulation involves a complex interplay of interactions, including inhibitory contacts between the KD and the regulatory SH3 and SH2 domains, coupled with conformational changes within the KD itself. These conformational states resulting from interdomain contacts are typically described as “open” and “closed”. The KD alternative conformations are classified by the most relevant contacts that occur within the KD as αC-in/out and DFG-in/out. Kinase activity requires the open, αC-in, and DFG-in conformations whereas the inactive forms may adopt distinct conformations. M305 chemical shifts primarily sense the αC state, but the closed state and the αC-out conformation of the KD are highly correlated. When interactions with SH3 and SH2 are destabilized or absent, KD is found in the αC-in form. On the other hand, drugs like eCF505 or A419259 forcing the αC-out conformation stabilize the closed conformation. This has the favorable consequence of inhibiting additional, nonphosphotransfer-dependent activities of Src.

### M305 Methyl ^13^C Chemical Shift Provides an Estimate of the Relative Importance of Different Regulatory Interactions

The M305 signal is the broadest peak in full-length Src in the absence of inhibitors, suggesting an intermediate exchange between alternative conformations. Upon binding of dasatinib or eCF506, the M305 signal sharpens, indicating a change in populations or the rate of conformational exchange. The observed chemical shift changes reflect these population shifts.

By using the M305 ^13^C chemical shift, we can estimate the effect of various regulatory interactions on the population of the active form of Src. The lowest population of the active form occurs when phosphorylated Y530 interacts with the SH2 domain. In unphosphorylated Src, the active form is populated at about 25%. Disruption of the SH3 domain interaction increases the population of the active state by 50%.

Phosphorylation of Y419 in the activation loop, in the absence of interaction between Y530 and the SH2 domain, increases the active state population by 75%. However, when pY530 binds to the SH2 domain, the population increase of the active state is only 25%. Similarly, phosphorylation of Y419 increases the population of the active form by 25% in the presence of the VSL12 peptide or in the isolated KD. Thus, a 25% population increase is associated with the effect on the KD itself, while phosphorylation of Y419 releases inhibitory contacts with the regulatory domains, which persist even in the absence of the pY530-SH2 interaction.

### Met-CH_3_ NMR Spectra Can Be Measured at Natural Abundance in Full-Length Src

**Figure 5:**
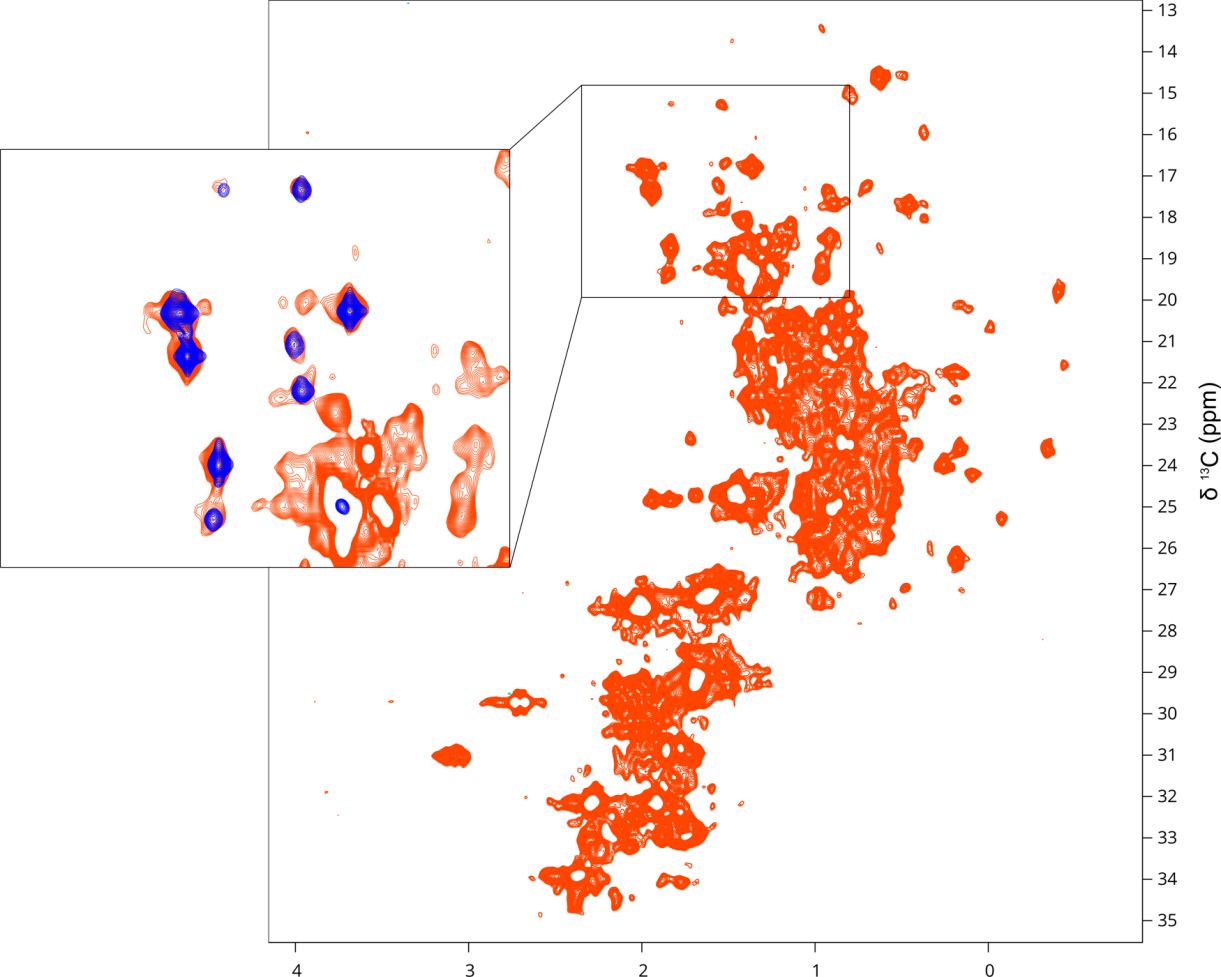
^1^H–^13^C XL-ALSOFAST HMQC spectrum of a 550 μM sample of full-length Src obtained at 298 K in a 1 GHz instrument. The expansion shows the Met-CH_3_ region superimposed with a spectrum from a Met-^13^CH_3_ enriched sample (blue).

The KD of all SFKs and other important tyrosine kinases, such as Abl, EGFR, EPH2, Met, or HER contains methionine residues in their KD.^29^ Those corresponding to M344, M377, and M498 in human Src are highly conserved. Thus, the use of Met-CH_3_ NMR should also be applicable to those kinases or other methionine-containing proteins. However, the use of isotopically labeled methionine for routine use would be restricted to proteins that can be expressed in good yields and purity in *E. coli*. This, in general is not trivial and therefore we explored the possibility of applying the same approach to natural abundance proteins measured at 1 GHz using ^1^H–^13^C XL-ALSOFAST HMQC.^30^Figure 5 compares the natural abundance spectrum of a 550 μM sample of full-length Src obtained in 8 h with that of a ^13^C-methyl methionine-enriched sample. Methionine signals appear to be well-resolved with minimal interference from other methyl signals. Spectra have a sufficient signal-to-noise ratio to detect even the weakest signals observed in the selectively labeled samples.

## Conclusion

Met-CH_3_ NMR provides information about the regulatory processes that control the activation state of Src. In the context of drug design, it offers information on the inhibition mechanism within hours, which can guide drug discovery efforts without the need to obtain X-ray quality crystals of complexes with new drug candidates.

Several methionine residues are conserved in other tyrosine protein kinases, making it feasible to extend this method to other important drug targets. The use of the recently developed XL-ALSOFAST HMQC, combined with the fact that methionine methyl signals show little overlap with other signals, enables the application of this method to proteins that cannot be selectively easily enriched with Met-^13^CH_3_.

Thus, this approach is an important addition to the drug discovery toolbox for key cancer-signaling proteins.

## Experimental Section

### KD Domain and Src Expression

Genes containing either the KD or full-length Src were synthesized by GenScript and subcloned in a pACYCDuet-1 dual plasmid in multiple cloning site 1 (MCS1). These genes contained an N-terminal His_6_-tagged SUMO fusion protein. The MCS2 contained the gene encoding the human Cdc37 cochaperone for the proper KD folding. The gene sequence of a GST-tagged YopH tyrosine phosphatase from *Yersinia enterocolitica* was also synthesized by GenScript and subcloned in a second pGEX-4T-1 plasmid. The Y530F mutant was also synthesized by GenScript.

Both plasmids were transformed in *E. coli* B834(DE3) competent cells, which are methionine auxotrophs, allowing the incorporation of an external methionine source provided in the culture media. A single colony from the Petri dish supplemented with chloramphenicol and ampicillin was transferred into 100 mL of an LB-based preculture containing the same antibiotics. The preculture was left shaking overnight at 37 °C. The following day, the preculture was centrifuged at 1000 *g* for 35 min at 4 °C. Then, the pellet was washed and resuspended in M9 minimal medium and transferred to 1 L of M9 minimal medium supplemented with 50 mg of l-methionine ^13^CH_3_, 99% (CLM-206–1, Cambridge Isotope Laboratories, Inc.). Cultures were grown at 37 °C until they reached an OD of 0.8, and expression was induced with IPTG for 30 min at the same temperature. The temperature was then reduced to 18 °C and the culture was left shaking overnight.

Cell pellets were harvested by centrifuging the 1 L cultures at 4000 *g* for 20 min at 4 °C and transferred into 25 mL of lysis buffer (50 mM Tris, pH = 8.0, 500 mM NaCl, 1 mM DTT and 0.01% NaN_3_) supplemented with 30 mM imidazole, 1 mM PMSF, and 1 mM benzamidine. Then, resuspended pellets were frozen at −80 °C.

For unlabeled samples, the 1 L culture was also LB-based, so that the overexpressed proteins consisted of natural abundance nuclei.

### Protein Purification

The frozen pellet contained in a 50 mL Falcon tube was thawed at room temperature and supplemented with 250 μL of lysozyme at 25 mg/mL and 100 μL of DNase I at 5 mg/mL. Cell disruption was accomplished by sonicating twice on ice at 80% of amplitude with 10 s on/off cycles for a total of 90 s. The lysate was clarified by centrifugation at 48,000 *g* for 40 min at 4 °C and then loaded onto a Ni-NTA column pre-equilibrated with lysis buffer supplemented with 30 mM imidazole. The column was extensively washed with the same buffer and eluted with lysis buffer containing 300 mM imidazole. Ulp1 protease was added in the elution fraction to cleave the N-terminal His-tagged SUMO fusion protein, followed by incubation for 1 h at room temperature. The reaction mixture was buffer exchanged into a solution containing 20 mM Tris, pH = 8.0, and 50 mM NaCl and injected into a 5 mL GSTrap HP (Cytiva) column to capture any residual GST-tagged YopH that may have coeluted in the first purification.

Next, the flowthrough from the GSTrap column was loaded into a 5 mL HiTrap Q HP (Cytiva) column to separate the cleaved His_6_-SUMO protein fusion from the Src construct. Fractions containing the constructs were applied to a Superdex 75 26/60 size exclusion column and buffer exchanged to 20 mM phosphate, pH = 7.5, 100 mM NaCl, and 0.01% sodium azide. For phosphorylated samples, 10 mM ATP and 20 mM MgCl_2_ were added to the elution from the ion exchange column, and phosphorylation was allowed to proceed overnight at room temperature before size-exclusion purification. Proteins were concentrated using Amicon Ultra-15 of 10 kDa MWCO (UFC9010, Sigma-Aldrich), flash-frozen in liquid nitrogen, and stored at −80 °C.

### NMR Samples and Experiments

For NMR experiments, 10 μM sodium 2,2-dimethyl- 2-silapentane-5-sulfonate (DSS) and 5% D_2_O or 5% DMSO-*d*_6_ were added for chemical shift referencing and locking purposes, respectively. The protein concentration was ca. 100 μM in 3 mm NMR tubes. The buffer consisted of 20 mM sodium phosphate, pH = 7.5, 100 mM NaCl, and 0.01% NaN_3_. Temperature was 298 K.

Dasatinib (99.88% purity), eCF506 (99.30% purity), ponatinib (99.43% purity), and A419259 (99.75% purity) were purchased from MedChemExpress and used without further purification.

Methionine assignments of the KD were performed on a Bruker 600 MHz Avance III, while the assignments involving full-length Src and all other measured methionine-labeled samples were performed on a Bruker 800 MHz Avance Neo. The natural abundance spectrum of Src was acquired on a Bruker 1 GHz Avance Neo. All spectrometers were equipped with TCI CryoProbes. The carbon dimensions in all spectra were indirectly referenced using DSS and the IUPAC-IUB recommended chemical shift referencing ratios (^13^C–^1^H = 0.251559530).

^1^H–^13^C SOFAST HMQC pulse sequence was used to acquire all Met-CH_3_ spectra of ^13^C-methionine-labeled full-length Src at 800 MHz. ^1^H selective pulses were centered at 1.5 ppm. Excitation was achieved by using a Pc9_4_120.1000 selective pulse with a length of 4.163 ms and a bandwidth of 2.1 ppm. The ^1^H-refocusing pulse was an Rsnob.1000 pulse with a length of 1.39 ms. The transfer delay (1/2*J*) was 3.62 ms (*J* = 138 Hz). The offset in proton was set to the water signal, while in carbon it was centered on the methionine signals (14.5 ppm). The number of scans was set to 128, the relaxation delay to 0.2 s, and acquisition time to 51 ms (interscan delay 0.251 s). The number of increments in the indirect dimension ranged from 80 to 150, depending on the spectral window containing all methyl groups from methionine residues, maintaining the same digital resolution.

^1^H–^13^C XL-ALSOFAST HMQC was used for the natural abundance Src unphosphorylated sample, measured at 1 GHz. The increments were 818 × 180, using a spectral window of 12.5 and 32 ppm for proton and carbon, respectively. The proton spectral window was centered on the water signal while carbon offset was set to 20 ppm for the carbon selective pulse (Reburp.1000). The number of scans was set to 300, giving a total experiment duration of 8 h. Optimized delays for this pulse sequence were τ1 = 2.66 ms, τ2 = 1.4 ms, and *D*1 = 0.5 s.

## Supporting Information Available

Spectra used for assignment and spectra in the presence of ponatinib and A419259 and chemical shift table (PDF)

## Abbreviations

KD: kinase domain
SFK: Src family kinases
