# Supplementary material for: A Fast Method to Monitor Tyrosine Kinase Inhibitor Mechanisms
Source: J Med Chem. 2024 Nov 8;67(22):20571–9. doi: 10.1021/acs.jmedchem.4c02042 (PMC11613495; doi:10.1021/acs.jmedchem.4c02042)
Supplement: Supplementary file 1 — jm4c02042_si_001.pdf [file jm4c02042_si_001.pdf]

Supporting information

A fast method to monitor tyrosine kinase inhibitor mechanisms

Alejandro Fernández,[1,3] Margarida Gairí,[2] María Teresa González,[2] Miquel Pons.[1]*

[1]Biomolecular NMR laboratory. Departament de Química Inorgànica i Orgànica. Universitat de Barcelona (UB). Baldiri Reixac 10-12, 08028 Barcelona. Spain.

[2] Centres Científics i Tecnològics de la Universitat de Barcelona (CCiTUB). Baldiri Reixac 10-12, 08028 Barcelona. Spain.

[3] PhD Program in Biotechnology, Faculty of Pharmacy. Universitat de Barcelona (UB), 08028 Barcelona, Spain.

Figure S1. Assignment spectra of Src's KD using site specific mutations

Figure S2. Assignment spectra for specific methionine residues in full-length Src

Figure S3. Spectra of full-length Src in the presence of ponatininb and A419259

Table ST1. Proton chemical shifts

Table ST2. Carbon-13 chemical shifts

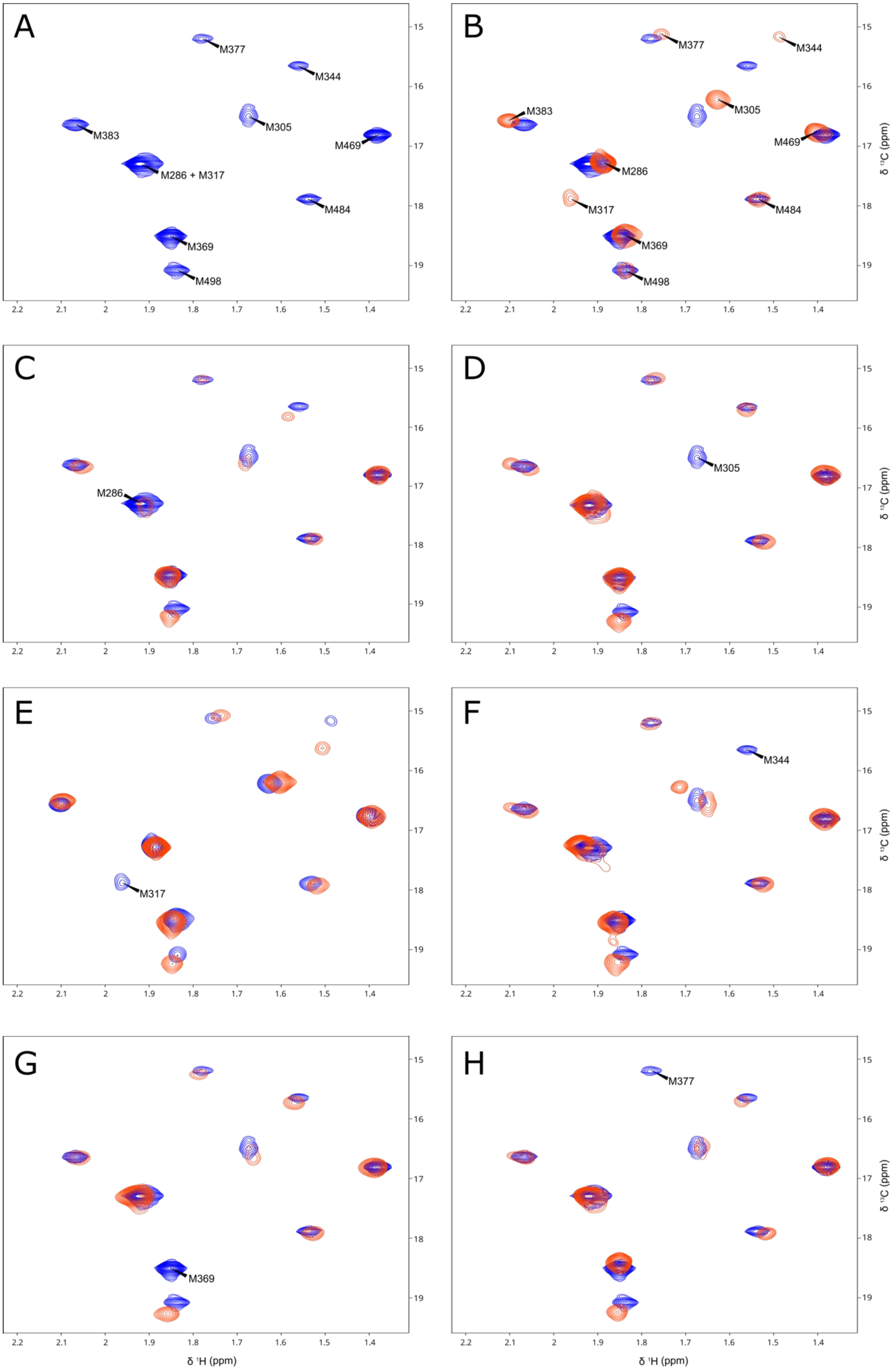
A
M377
M344
M305
M383
M469
M286 + M317
M484
M369
M498
B
M377
M344
M383
M305
M469
M286
M317
M484
M369
M498
C
M286
D
M305
E
M317
F
M344
G
M369
H
M377
δ ¹H (ppm)
δ ¹³C (ppm)

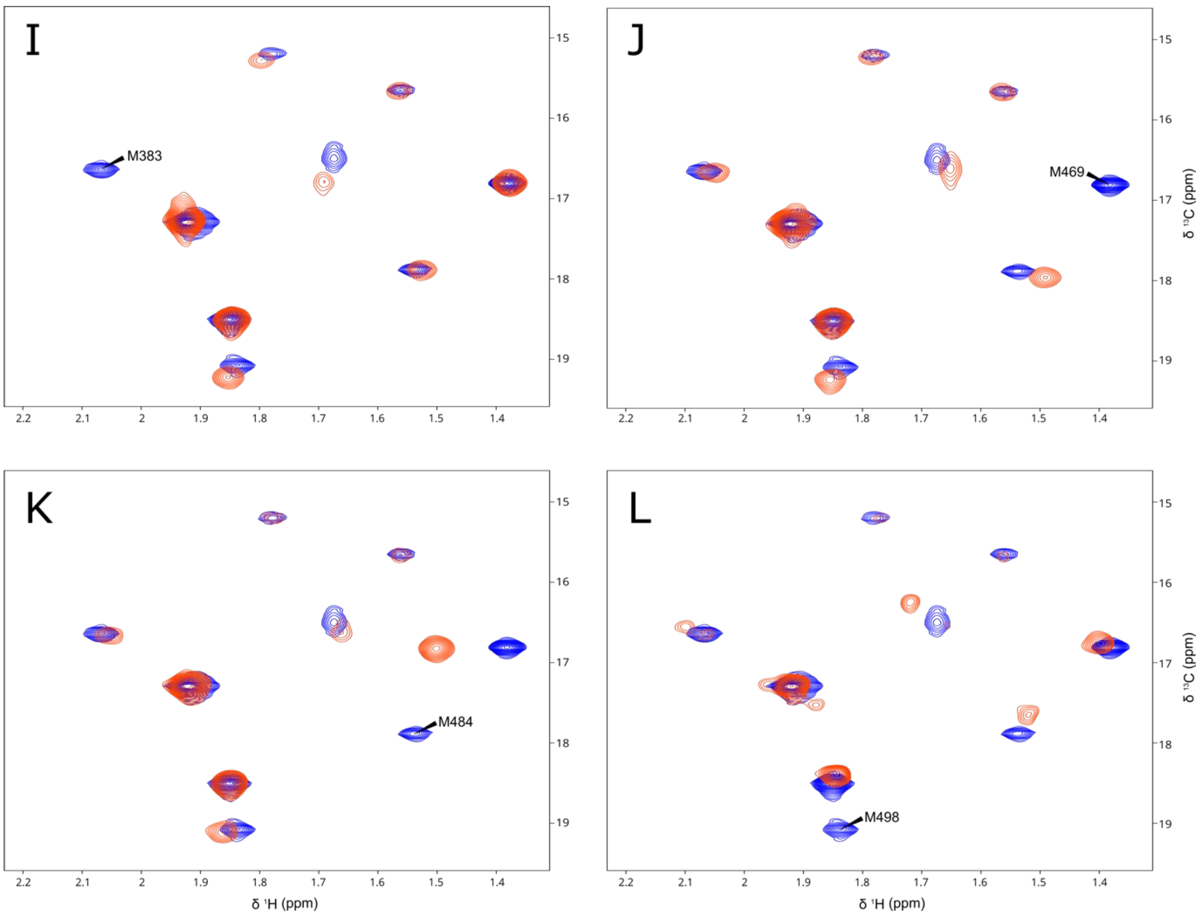

Figure S1. KD assignment. (A) WT spectrum with assignments. A reference spectrum is reproduced (blue) in all panels for direct comparison with the spectra of the perturbed systems (red). (B) WT plus ATP; (C) M286I; (D) M305L; (E) M317L plus ATP. In this panel the reference spectrum is (B); (F) M344L; (G) M369L; (H) M377L; (I) M383L; (J) M469L; (K) M484L; (L) M498L.

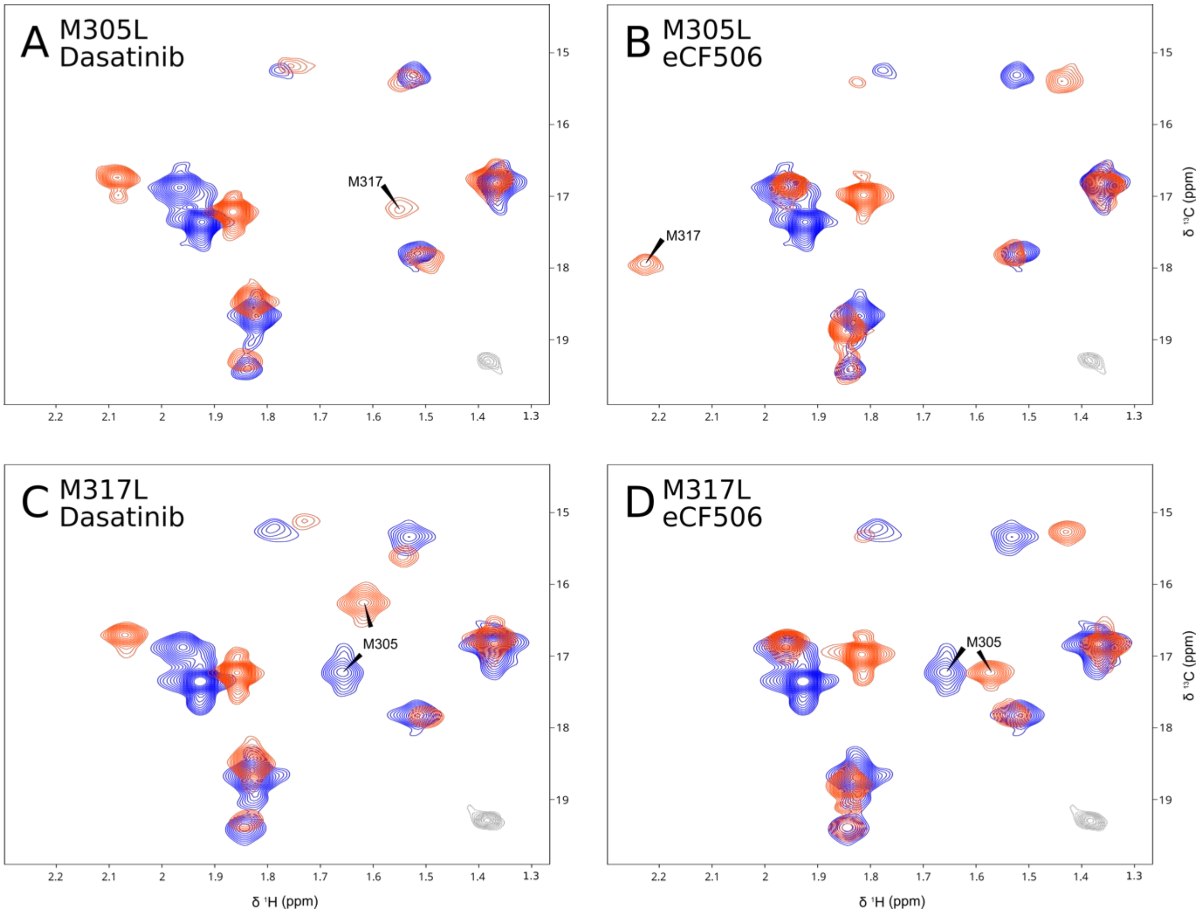

Figure S2. Assignment of full-length Src drug complexes. (A) [M305L] Src before (blue) and after (red) the addition of dasatinib; (B) [M317L] Src before (blue) and after (red) the addition of eCF505; (C) [M317L] Src before (blue) and after (red) the addition of dasatinib; (D) [M317L] Src before (blue) and after (red) the addition of eCF505. Notice the very large shifts of M317 caused by eCF505 and the accidental near coincidence of the signal from M317 in the presence of dasatinib with that of M305 in full length Src in the presence of eCF505 or in the absence of inhibitors (cf. Figure 1).

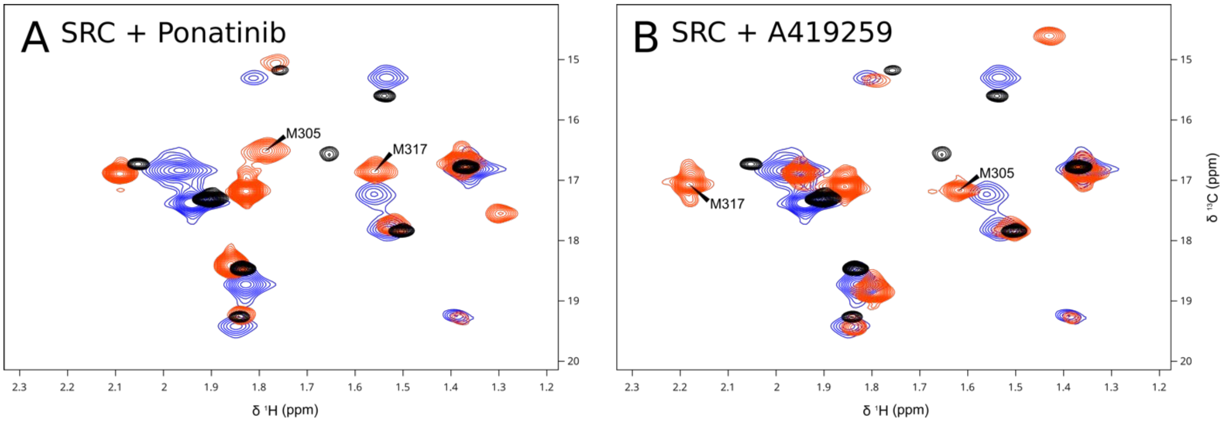

Figure S3. Met-$^{13}CH_3$ spectra of full-length Src complexes with ponatininb (A) and A419259. A reference spectrum of Src without added drugs is shown in blue.

Table ST1. Proton chemical shifts (ppm)

Sample	M286	M305	M317	M344	M369	M377	M383	M469	M484	M498
KD	1.908	1.654	-	1.539	1.834	1.757	2.053	1.370	1.504	1.842
pKD	1.901	1.695	1.878	1.531	1.837	1.742	2.087	1.389	1.502	1.842
Src	1.926	1.577	-	1.529	1.823	1.793	1.965	1.364	1.518	1.841
pSrc	1.932	1.659	-	1.528	1.816	1.787	1.965	1.390	1.521	1.851
Y530FSrc	1.938	1.558	-	1.530	1.825	1.805	1.963	1.366	1.529	1.845
Y530FpSrc	1.906	1.694	1.897	1.534	1.843	1.748	2.096	1.392	1.507	1.848
Src +VSL12	1.913	1.661	-	1.548	1.838	1.772	2.043	1.369	1.515	1.842
pSrc +VSL12	1.910	1.710	-	1.526	1.835	1.743	2.078	1.396	1.509	1.851
Src +dasatinib	1.861	1.588	1.544	1.533	1.825	1.753	2.081	1.371	1.495	1.839
Src + eCF506	1.808	1.537	2.189	1.431	1.842	1.818	1.949	1.354	1.527	1.840

Table ST2. Carbon chemical shifts (ppm)

Sample	M286	M305	M317	M344	M369	M377	M383	M469	M484	M498
KD	17.316	16.576	-	15.602	18.479	15.179	16.731	16.779	17.846	19.263
pKD	17.324	16.318	17.549	15.627	18.474	15.141	16.655	16.748	17.879	19.269
Src	17.367	17.173	-	15.376	18.674	15.282	16.892	16.849	17.821	19.355
pSrc	17.320	17.620	-	15.222	18.683	15.216	16.825	16.694	17.713	19.863
Y530FSrc	17.375	17.250	-	15.266	18.689	15.271	16.828	16.786	17.766	19.652
Y530FpSrc	17.308	16.294	17.571	15.597	18.445	15.100	16.627	16.693	17.843	19.495
Src +VSL12	17.311	16.674	-	15.605	18.495	15.144	16.740	16.768	17.826	19.242
pSrc +VSL12	17.320	16.265	-	15.580	18.503	15.066	16.696	16.666	17.772	19.817
Src +dasatinib	17.241	16.160	17.148	15.365	18.454	15.149	16.708	16.759	17.835	19.268
Src + eCF506	16.966	17.192	17.925	15.365	18.853	15.422	16.831	16.837	17.790	19.423
